# Ghrelin receptor agonist MK0677 and overnight fasting do not rescue deficient fear extinction in 129S1/SvImJ mice

**DOI:** 10.3389/fpsyt.2023.1094948

**Published:** 2023-02-09

**Authors:** Eva Maria Fritz, Anouk Pierre, Dimitri De Bundel, Nicolas Singewald

**Affiliations:** ^1^Department of Pharmacology and Toxicology, Institute of Pharmacy and CMBI, University of Innsbruck, Innsbruck, Austria; ^2^Department of Pharmaceutical Sciences, Research Group Experimental Pharmacology, Center for Neurosciences (C4N), Vrije Universiteit Brussel, Brussels, Belgium

**Keywords:** ghrelin, GHSR agonist, impaired fear extinction, 129S1/SvImJ mice, fasting, MK0677

## Abstract

The hunger hormone ghrelin has been implicated in the modulation of anxiety- and fear-related behaviors in rodents and humans, while its dysregulation may be associated with psychiatric illness. Along these lines, the ghrelin system has been suggested as a potential target to facilitate fear extinction, which is the main mechanism underlying cognitive behavioral therapy. So far, this hypothesis has not been tested in individuals that have difficulties to extinguish fear. Thus, we investigated pharmacological (ghrelin receptor agonist MK0677) and non-pharmacological (overnight fasting) strategies to target the ghrelin system in the 129S1/SvImJ (S1) mouse strain, which models the endophenotype of impaired fear extinction that has been associated with treatment resistance in anxiety and PTSD patients. MK0677 induced food intake and overnight fasting increased plasma ghrelin levels in S1 mice, suggesting that the ghrelin system is responsive in the S1 strain. However, neither systemic administration of MK0677 nor overnight fasting had an effect on fear extinction in S1 mice. Similarly, our groups previously reported that both interventions did not attenuate fear in extinction-competent C57BL/6J mice. In summary, our findings are in contrast to several studies reporting beneficial effects of GHSR agonism and overnight fasting on fear- and anxiety-related behaviors in rodents. Rather, our data agree with accumulating evidence of divergent behavioral effects of ghrelin system activation and underscore the hypothesis that potential benefits of targeting the ghrelin system in fear extinction may be dependent on factors (e.g., previous stress exposure) that are not yet fully understood.

## 1. Introduction

The notion of ghrelin as an important junction of metabolism, stress, mood and reward (1) has raised interest concerning its role in psychiatric conditions like addiction, depression, anxiety disorders and post-traumatic stress disorder (PTSD) (2, 3). These disorders are often associated with metabolic dysregulation and show a high level of comorbidity with obesity and eating disorders as well as with each other (4, 5). This suggests a common neurobiological pathology, which represents an interesting possibility to study potential diagnostic biomarkers and novel therapeutic targets (6). Dysregulation of the ghrelin system in psychiatric disorders, and anxiety- and trauma-related disorders in particular, has been reported in several studies. For example, polymorphisms in the pre-proghrelin gene have been linked to an increased risk of panic disorder (7) or symptom severity in PTSD (8). Some studies reported altered plasma ghrelin levels in different anxiety disorders (9–11), while a recent population-based trial found that total serum ghrelin levels were positively associated with physiological anxiety, but negatively associated with pathological anxiety (12). Also, one study reported that therapy resistance in panic disorder and depression was correlated with elevated plasma acyl ghrelin (11). However, to date, it remains unclear whether a modulation of the ghrelin system could be beneficial in the treatment of anxiety- and trauma-related disorders (6). Several rodent studies point to a positive effect of ghrelin receptor (GHSR) agonism on fear- and anxiety-like behaviors (13–17). Moreover, accumulating evidence from preclinical studies suggests that caloric restriction or short-term fasting can reduce anxiety-like behaviors and attenuate fear, probably in a ghrelin-dependent manner (13, 18–21). Interestingly, also in healthy human participants overnight fasting before a fear extinction session reduced the return of fear, which was correlated with plasma ghrelin levels (22). Along these lines, it appears as a particularly interesting therapeutic avenue to target the ghrelin system as an adjunct to psychotherapy to improve treatment efficacy: While cognitive behavioral therapy, which relies on fear extinction, is a gold-standard treatment for anxiety disorders and PTSD, many patients experience only limited treatment success and relapse after time (23, 24), which is likely due to individual deficits in the ability to acquire, consolidate or retrieve extinction memories (25, 26). However, so far, it has not been studied whether GHSR agonism or short-term fasting can rescue deficient fear extinction. One relevant rodent model to investigate this, is the 129S1/SvImJ mouse strain, which exhibits severe deficits in Pavlovian fear extinction paradigms (25, 27, 28). In the current study, we tested the functionality of the ghrelin system in S1 mice compared to the well-characterized responses of C57BL/6J (BL6) mice to GHSR agonist administration and short-term fasting and investigated GHSR agonist MK0677 and overnight fasting as potential means to attenuate fear in the S1 mouse strain. Because we previously found no effect of MK0677 administration or fasting on fear extinction in BL6 mice (29), we did not include additional BL6 groups in fear conditioning and extinction experiments in this study.

## 2. Methods

### 2.1. Animals and husbandry

All experiments were carried out in compliance with national and international guidelines for animal welfare and as approved by the Austrian Federal Ministry of Science and Research (BMWFW-66.008/0019-WF/V/3b/2016, BMBWF-66.008/0041-WF/V/3b/2019). Male, adult 129S1/SvlmJ (S1) mice and C57BL/6J (BL6) mice (10–16 weeks at start of experiments) were bred in-house or purchased from Charles River as indicated (Charles River Laboratories, Sulzfeld, Germany). The animals were housed under constant conditions (22 ± 2^°^C, 40–60% rH, lights on/off at 7 a.m./7 p.m.) with *ad libitum* access to food and water unless stated otherwise. All mice were held in groups (max. five per cage) and single housed 3 days before start of the experiments to be able to measure food consumption and prevent possible aggression during fasting procedures or after injection of MK0677. The number of mice (n) for each experimental group are given in the corresponding figure legends.

### 2.2. Drug administration and overnight fasting

MK0677 (Sigma-Aldrich/Merck, Darmstadt, Germany) was dissolved in sterile 0.9% saline (Braun, Melsungen, Germany) and administered intraperitoneally (i.p.) at increasing doses of 2, 5,and 10 mg/kg as stated in the corresponding figure legends. During behavioral experiments, MK0677 was administered 1 h before extinction training. For overnight fasting (ONF), the mice were transferred to fresh cages before onset of the dark period to ensure that no food remained in the cages, while control animals were placed into fresh cages with *ad libitum* access to food. During behavioral experiments, the animals were fasted 15–18 h before each extinction training session, while every other day and night the mice were re-fed.

### 2.3. Quantification of eating response

Before the start of experiments, all animals were habituated to the handling procedure for 3 days. Pre-weighed food pellets were placed in the food grid of the home cages and overnight food consumption was measured in the morning (7.30 a.m.). A pre-weighed amount of food pellets was introduced again (08:00 a.m.) and video recordings were started to observe the eating behavior of the animals for 90 min at baseline and after i.p. injection of either sterile 0.9% saline or MK0677 (10 mg/kg). The remaining food pellets were weighed before injection and at the end of the experiment. Time spent eating was manually scored and the distance traveled automatically tracked with ANY-maze behavioral tracking software (Stoelting Europe, Dublin, Ireland).

### 2.4. Total and acyl ghrelin ELISA assays

Trunk blood was collected from overnight fasted and non-fasted S1 and BL6 mice in EDTA-coated blood collection tubes (Minicollect, greiner bio-one, Kremsmünster, Austria). Pefabloc (Sigma-Aldrich/Merck, Darmstadt, Germany) stock solution (100 mg/ml in distilled H2O) was added to a concentration of 1 mg/ml blood. The samples were then centrifuged at 2,000 rpm, the supernatant transferred to a fresh sample tube and 0.5 N HCl was added to a final concentration of 0.05 N. The plasma samples were stored at −20^°^C for later analysis. If samples showed signs of hemolysis they were not processed for analysis. Plasma levels for acyl and total ghrelin levels were measured with ready-to-use ELISA kits (#EZRGRA-90K Rat/Mouse Active Ghrelin ELISA Kit and #EZRGRA-91K Rat/Mouse Total Ghrelin ELISA Kit, Merck Millipore, Darmstadt, Germany) according to the manufacturer’s instructions. All samples were loaded in duplicates with a sample size of 20 μl. The standard curve was plotted as a sigmoidal four-parameter logistic function, based on which the concentration in all unknown samples was calculated. For assay characteristics, please see Supplementary material.

### 2.5. Fear conditioning and extinction

The behavioral protocol was adapted from Verma et al. (20). Before the start of experiments, all animals were habituated to the handling procedure for 3 days. Fear conditioning and extinction procedures were controlled by a TSE operant system (TSE, Bad Homburg, Germany). All sessions were video recorded for later analysis.

The animals were fear conditioned (FC, day 1, context A) using five pairings of a conditioned stimulus (CS, 10 kHz sine tone, 75 dB, 30 s) with a co-terminating unconditioned stimulus (US, scrambled foot shock, 0.6 mA, 2 s). On the next day, all mice underwent extinction training (EXT, context B) with 25 CS-only presentations. 24 h later, extinction memory was tested in a retrieval session (RET, context B) with five CS presentations. In total, three cycles of EXT-RET were performed. After 2 weeks, fear reinstatement (REIN, context B) was tested 24 h after the delivery of an unsignaled US in context A. Freezing behavior [i.e., absence of any movement except for respiration (30)] was either manually scored with a custom-written MATLAB script (Mathworks, Natick, MA, USA) or automatically detected with ANY-maze behavioral tracking software according to pre-defined thresholds that were validated by manual scoring. For details, please see Supplementary material.

### 2.6. Statistical analysis

GraphPad Prism 9 (GraphPad Software, San Diego, CA, USA) software was used for generating graphs and performing statistical analysis. All data sets were tested for normality and two-tailed, unpaired or paired Student’s *t*-test and Mann-Whitney test were used for two group comparisons of normally and not normally distributed data, respectively. Experiments with two independent variables were analyzed by a two-way ANOVA or two-way repeated measures (RM) ANOVA if one of the variables was time. A Tukey *post-hoc* test was applied where multiple comparisons were of interest. The significance level was set to *p* < 0.05 (^*^p < 0.05, ^**^p < 0.01, ^***^p < 0.001, ^****^p < 0.0001). All data are presented as individual data points and/or group means ± SEM unless stated otherwise. For detailed statistics, please see Supplementary Tables 1–3.

## 3. Results

### 3.1. Systemic MK0677 administration increases food intake in S1 and BL6 mice

Food consumption in S1 and BL6 mice was monitored overnight, 90 min prior to (stage: baseline) and after administration (stage: post-injection) of either MK0677 (S1 MK0677, *n* = 8; BL6 MK0677, *n* = 7) or saline (S1 saline, *n* = 8; BL6 saline: *n* = 8). One animal from the BL6 MK0677 group was excluded from all analyses because it showed abnormal behavior at baseline (extensive digging and hyperlocomotion), which was reflected as an outlier the distance traveled (89.22 m). Overnight and baseline data were pooled per strain, as there was no significant difference between the groups pre-treatment (Supplementary Table 1).

**FIGURE 1:**
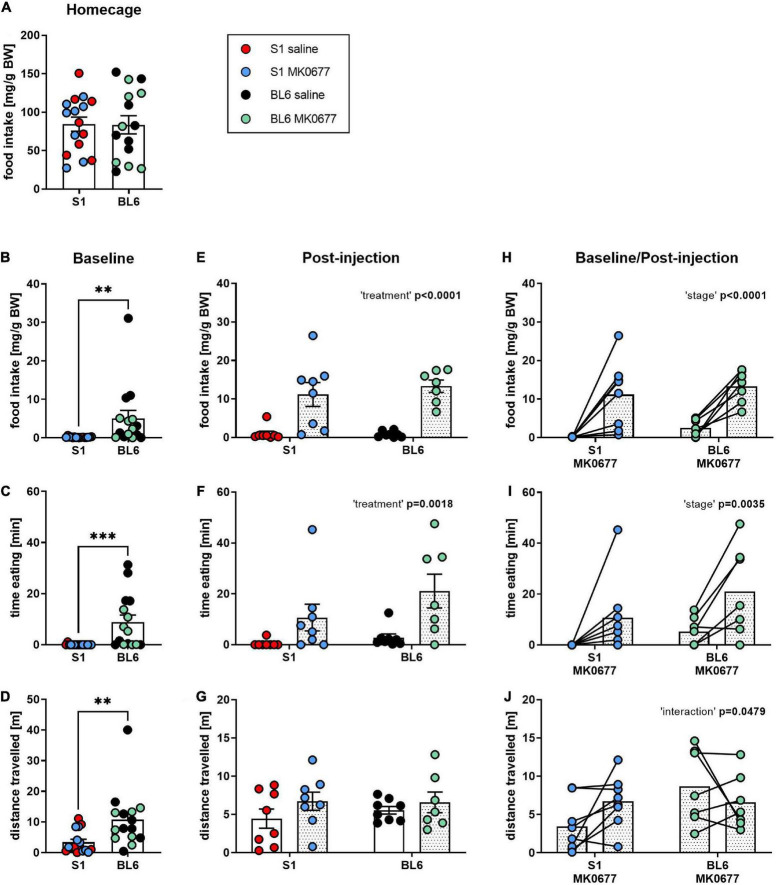
Eating response to a single injection of ghrelin receptor (GHSR) agonist MK0677 intraperitoneally (i.p., 10 mg/kg) in 129S1/SvImJ (S1) and C567BL/6J (BL6) mice. **(A)** Overnight (16 h) home cage food intake. Overnight food intake did not differ between the strains (*p* = 0.9580). **(B–D)** Baseline (90 min) food intake, time spent eating and distance traveled. At baseline, food intake (^**^p = 0.0021), time spent eating (^***^p = 0.0001) and distance traveled (^**^p = 0.0023) were significantly higher in BL6 than S1 mice **(E–G)**. Post-injection (90 min) food intake, time spent eating and distance traveled. Mice of both strains showed an eating response to MK0677 but not saline injection (food intake, time eating: “treatment” *p* < 0.0001, F (1, 27) = 40.04, ^**^p = 0.0018, F (1, 27) = 11.98). The distance traveled was comparable between S1 and BL6 animals of both treatments. **(H–J)** Change of food intake, time spent eating and distance traveled after MK0677 injection. MK0677 caused a significant increase in food intake [“stage” *p* < 0.0001, F (1, 13) = 35.08] and time spent eating [“stage” ^**^p = 0.0035, F (1, 13) = 12.70] regardless of strain. “Strain” × “treatment” interaction for distance traveled reached significance [“interaction” ^*^p = 0.0479, F (1, 13) = 4.769], but a Tukey *post-hoc* test was not significant (baseline vs. post-injection for S1 MK0677, BL6 MK0677: *p* = 0.1420, *p* = 0.4564). Figure info: S1 MK0677 (blue): *n* = 8; S1 saline (red): *n* = 8; BL6 MK0677 (green): *n* = 7; BL6 saline (black): *n* = 8. All data points are individual values. All bars represent group means ± SEM. BW, bodyweight.

Overnight food intake in the home cage did not differ between both strains (Figure 1A). In the 90 min before injection, however, baseline food intake (Figure 1B, Mann-Whitney test: *p* = 0.0021) and time spent eating (Figure 1C, Mann-Whitney test: *p* = 0.0001) as well as the mean distance traveled (Figure 1D, Mann-Whitney test: *p* = 0.0023) were lower in S1 than BL6 mice. The injection of MK0677 increased food intake (Figure 1E, Two-way ANOVA: “treatment” *p* < 0.0001) and time spent eating (Figure 1F, Two-way ANOVA: “treatment” *p* = 0.0018) compared to saline-treated animals regardless of strain. The distance traveled after injection, however, did not significantly differ between strains and treatments (Figure 1G). The increase in food intake (Figure 1H, Two-way RM ANOVA: “stage” *p* < 0.0001) and time spent eating (Figure 1I, Two-way RM ANOVA: “stage” *p* = 0.0035) from baseline after MK0677 administration was significant, while there was no effect of strain. For the distance traveled, the “strain” × “treatment” interaction was significant (Figure 1J, Two-way RM ANOVA: “interaction” *p* = 0.0479), however, a multiple comparisons *post-hoc* test did not deliver significant results (Tukey *post-hoc* test (baseline vs. post-injection) for S1 MK0677, BL6 MK0677: *p* = 0.1420, *p* = 0.4564).

### 3.2. Overnight fasting induces ghrelin release in S1 and BL6 mice

Plasma acyl and total ghrelin levels of overnight fasted S1 and BL6 mice (S1 ONF, *n* = 8; BL6 ONF, *n* = 8) and controls with *ad libitum* access to food (S1 non-ONF, *n* = 9; BL6 non-ONF, *n* = 7) were measured using ELISA assays.

**FIGURE 2:**
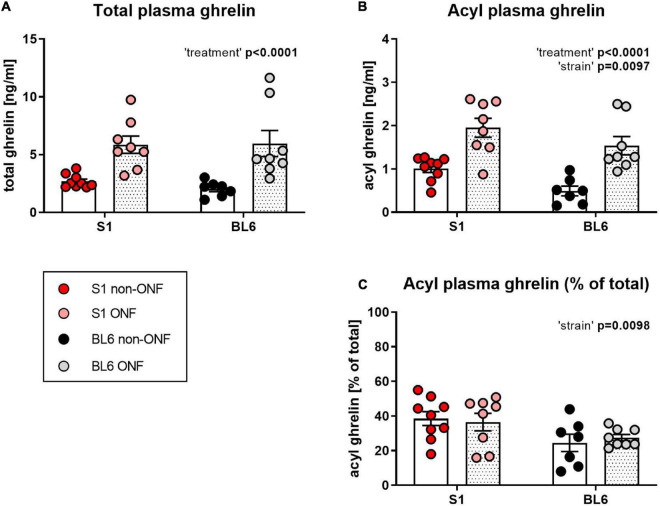
Plasma ghrelin levels of overnight fasted and not fasted 129S1/SvImJ (S1) and C567BL/6J (BL6) mice. **(A)** Total ghrelin plasma levels. Regardless of strain, total ghrelin levels were increased in fasted mice [“treatment” *p* < 0.0001, F (1, 28) = 25.52]. **(B)** Acyl ghrelin plasma levels. Acyl ghrelin levels were increased in fasted mice of both strains, but higher in S1 than BL6 mice regardless of feeding status [“treatment” *p* < 0.0001, F (1, 28) = 34.72; “strain” *p* = 0.0097, F (1, 28) = 7.716]. **(C)** Percentage of acyl ghrelin of total plasma ghrelin. Acyl ghrelin percentage was not influenced by fasting, but higher in S1 than in BL6 mice [“strain” *p* = 0.0098, F (1, 28) = 7.672]. Figure info: S1 ONF (pink): *n* = 8; S1 non-ONF (red): *n* = 9; BL6 ONF (gray): *n* = 8; BL6 non-ONF (black): *n* = 7. All data points are individual values. All bars represent group means ± SEM. ONF, overnight fasted.

Total plasma ghrelin levels were comparable between S1 and BL6 mice and increased by fasting (Figure 2A, Two-way ANOVA: “treatment” *p* < 0.0001). Acyl ghrelin levels were also elevated after fasting in both strains, however, they were higher in S1 than BL6 mice (Figure 2B, Two-way ANOVA: “treatment” *p* < 0.0001; “strain” *p* = 0.0097). The percentage of acyl ghrelin of total plasma ghrelin was higher in S1 than BL6 mice regardless of feeding status, but not changed by fasting (Figure 2C, Two-way ANOVA: “strain” *p* = 0.0098).

### 3.3. Systemic MK0677 administration has no effect on fear extinction in S1 mice

**FIGURE 3:**
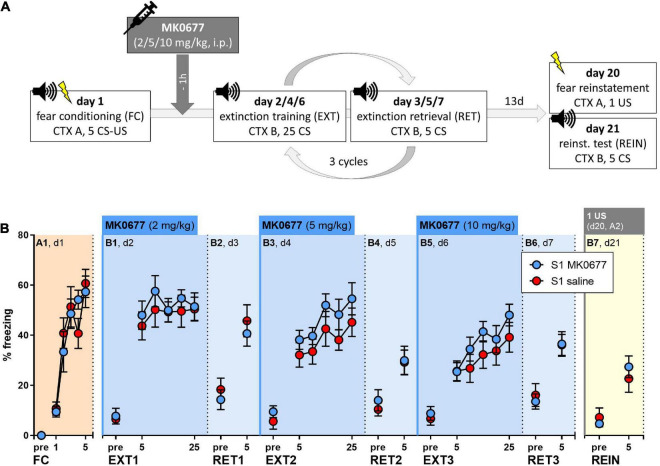
Effect of pre-extinction training administration of MK0677 intraperitoneally i.p., 2 mg/kg (d2, EXT1), 5 mg/kg (d4, EXT2), or 10 mg/kg (d6, EXT3) on fear extinction in 129S1/SvImJ (S1) mice. **(A)** Experimental protocol and conditions for all behavioral tests. **(B)** Percent of time spent freezing during pre-CS periods or indicated CS blocks throughout the experimental paradigm. Both groups showed fear acquisition (FC/A1: “CS block” *p* < 0.0001, F (3.537, 49.52) = 27.27) and no significant differences during extinction training (EXT1/B1, EXT2/B3, EXT3/B5), extinction retrieval or a fear reinstatement test (RET1/B2, RET2/B4, RET3/B6, REIN/B7). Figure info: S1 MK0677 (blue): *n* = 8; S1 saline (red): *n* = 8. All data points represent group means ± SEM for the indicated time periods (pre-CS, single CS, or 5-CS average blocks). FC, fear conditioning; EXT, extinction training; RET, extinction retrieval; REIN, fear reinstatement test; US, unconditioned stimulus; CS, conditioned stimulus; pre, pre-CS habituation.

129S1/SvImJ mice were fear conditioned (FC, A1, day 1) and exposed to three cycles of extinction training (EXT, B1/B3/B5, days 2/4/6) and extinction memory retrieval (RET, B2/B4/B6, days 3/5/7) (Figure 3A). One hour before each extinction training session, the mice were i.p. injected with either increasing doses of MK0677 (2, 5 or 10 mg/kg, days 2/4/6); S1 MK0677, *n* = 8) or sterile saline (S1 saline, *n* = 8). Fear reinstatement was tested 2 weeks later (REIN, B7, day 21), after exposure to a single, unsignaled foot shock in the fear conditioning context 1 day before (REIN, A2, day 20) (Figure 3A).

Both groups responded to fear conditioning with an increase in freezing levels across trials (Figure 3B, Two-way RM ANOVA for A1: “CS block” *p* < 0.0001). On the following days, pre-extinction administration of MK0677 had no significant effect on within-session extinction (Figure 3B) or extinction retrieval (Figure 3B). Within-session fear incubation was evident during extinction training on days 4 and 6 (Figure 3B, Two-way RM ANOVA for B3, B5: “CS block” *p* = 0.0019, *p* < 0.0001). There were no differences in freezing levels between the groups during the fear reinstatement test (Figure 3B). Notably, following the delivery of one US in context A, we did not observe an increase but rather a decrease in freezing levels regardless of treatment (Supplementary Figure 1, Two-way RM ANOVA: “CS block” *p* < 0.0001).

### 3.4. Overnight fasting does not rescue the extinction-impaired phenotype of S1 mice

**FIGURE 4:**
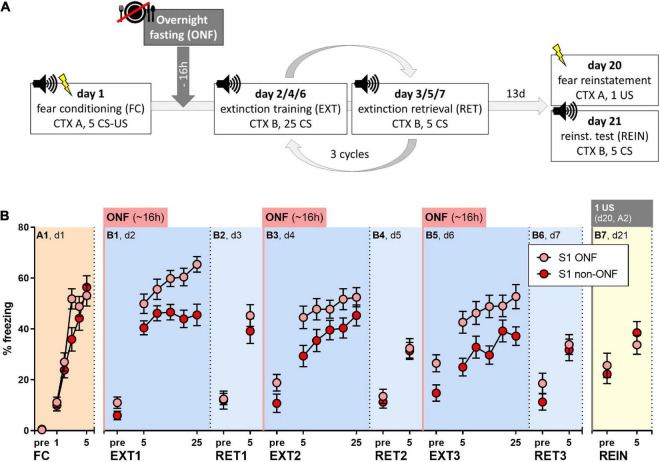
Effect of pre-extinction training overnight fasting on fear extinction in 129S1/SvImJ (S1) mice. **(A)** Experimental protocol and conditions for all behavioral tests. **(B)** Percent of time spent freezing during pre-CS periods or indicated CS presentations throughout the experimental paradigm. Both groups showed sufficient fear acquisition [FC/A1: “CS block” *p* < 0.0001, F (3.542, 159.4) = 56.56]. Overnight fasting before fear extinction increased freezing levels compared to non-fasted controls [“treatment” for EXT1/B1; EXT2/B3; EXT3/B5: *p* = 0.0011, F (1, 45) = 12.11; *p* = 0.0346, F (1, 45) = 4.749, *p* = 0.0052, F (1, 45) = 8.618], while there were no significant differences during extinction retrieval or fear reinstatement tests when all mice were fed (RET1/B2, RET2/B4, RET3/B6, REIN/B7). Figure info: S1 ONF (pink): *n* = 23; S1 non-ONF (red): *n* = 24. All data points represent group means ± SEM for the indicated time periods (pre-CS, single CS or 5-CS average blocks). ONF, overnight fasted; FC, fear conditioning; EXT, extinction training; RET, extinction retrieval; REIN, fear reinstatement test; US, unconditioned stimulus; CS, conditioned stimulus; pre, pre-CS habituation.

All behavioral procedures were carried out analogously to experiments in 3.3 (Figure 4A). Before each extinction training session, the mice were either overnight fasted for 15–18 h (S1 ONF, *n* = 23) or had *ad libitum* access to food (S1 non-ONF, *n* = 24). The data presented here were pooled from two different sets of experiments with mice bred in-house or purchased from Charles River. One mouse in the S1 ONF group was excluded from analysis because not all foot shocks were delivered during fear conditioning.

Both groups showed comparable fear acquisition (Figure 4B, Two-way RM ANOVA for A1: “CS block” *p* < 0.0001). On the following days, overnight fasting did not facilitate within-session extinction, but rather increased freezing in fasted mice (Figure 4B, Two-way RM ANOVA for B1, B3, B5: “treatment” *p* = 0.0011, *p* = 0.0346, *p* = 0.0052), while both groups showed within-session fear incubation (Figure 4B, Two-way RM ANOVA for B1, B3, B5: “CS block” *p* < 0.0001, *p* < 0.0001, *p* < 0.0001). During extinction retrieval sessions, when all mice were fed, freezing levels of fasted and non-fasted S1 mice were similar (Figure 4B). This was also the case during the fear reinstatement test (Figure 4B), where, again, we did not observe increased freezing levels (Supplementary Figure 1).

## 4. Discussion

Many studies in rodents suggest that ghrelin and the GHSR play a role in the regulation of fear and anxiety-like behaviors. Which role, however, is still a matter of debate. We previously reported that a loss of GHSR function, induced either *via* exposure to a high-fat diet (31) or a knock-out of GHSR, had no significant effect on fear extinction or anxiety-like behaviors in mice on a BL6 background (32). This led us to conclude that intact GHSR signaling is no pre-requisite to extinguish fear and that GHSR dysfunction *per se* has no influence on fear and anxiety-related behaviors (32). Nevertheless, this did not exclude the possibility that a manipulation of the ghrelin system could have anxiolytic or fear-reducing effects, as suggested by several rodent studies [for summary see (6)]. In the current study, we investigated for the first time whether GHSR agonism and overnight fasting would be sufficient interventions to rescue the extinction-impaired phenotype of the S1 mouse strain.

Before testing whether an activation of the ghrelin system can ameliorate the extinction-impaired phenotype of the S1 strain, we wanted to confirm its functionality in comparison to the responses of (typically extinction-competent) BL6 mice to GHSR agonism and overnight fasting. In our experiments, the GHSR agonist MK0677 induced food intake in S1 similar to BL6 mice (but had no effect on locomotion that could potentially confound behavioral readouts), while overnight fasting stimulated ghrelin release in both mouse strains, suggesting a responsive ghrelin system in the S1 line. Interestingly, while MK0677 increased food intake in both, S1 and BL6 mice, baseline food intake shortly before injection of saline or MK0677 was lower in S1 mice. Overnight food intake prior to the experiment, on the other hand, was similar in both mouse strains. The circadian distribution of wakefulness and sleep is strongly altered in S1 compared to BL6 animals (33), thus, it is likely that also the peak times of food intake may differ between the strains. This is furthermore corroborated by the finding that the baseline distance traveled was lower in S1 than BL6 mice, which indicates decreased home cage activity at the beginning of the light period. In agreement with this finding, reduced spontaneous locomotion and home cage activity in the S1 strain has been reported before (34–36). We did not observe spontaneous freezing in the S1 animals (data not shown, but also see no pre-CS habituation during fear conditioning) as food intake measurements were conducted in the home cage to minimize anxiogenic effects during the test. Importantly, it should be considered that alterations in circadian rhythms may not only influence food intake but also ghrelin secretion. Overnight fasting stimulated ghrelin release in both mouse strains, however, we found elevated acyl ghrelin levels in S1 compared to BL6 mice regardless of their feeding status. Ghrelin secretion in rodents follows a diurnal rhythm in rodents (37, 38), therefore we cannot exclude that these differences in strain are merely a byproduct of different circadian sleep/wake behaviors in S1 and BL6 lines.

Interestingly, elevated plasma acyl ghrelin has been suggested as a persistent biomarker for chronic stress exposure that mediates vulnerability to stress enhanced fear in a translational study (39, 40) and another study in patients found that therapy resistance in panic disorder and depression was correlated with increased acyl ghrelin levels (11). While stress (and chronic stress in particular) increases the acyl ghrelin concentration in the blood (13, 41–43), it should be taken into consideration that chronically elevated plasma acyl ghrelin at baseline may also represent a feature of individuals that are particularly susceptible for the development of stress-related behaviors or psychiatric disorders. Indeed, some studies found elevated (acyl) plasma ghrelin levels in different anxiety disorders (9–11). A recent population-based cohort study reported that total ghrelin levels were negatively associated with pathological anxiety, but (non-significantly) higher in subjects with clinically relevant anxiety symptoms than in those without (12). Generally, these findings suggest that ghrelin plays a role in the regulation of fear and anxiety-like behaviors and/or is altered by either previous stress exposure or generally elevated in stress-susceptible individuals. Along these lines, it seems plausible that impaired fear extinction, which was proposed as a risk factor for treatment resistance in anxiety and PTSD patients (44, 45), could also be associated with elevated acyl ghrelin levels. In our study, we did not directly address this hypothesis by correlating behavioral parameters of extinction-impaired S1 mice with plasma acyl ghrelin levels, so it remains to be tested in (other) rodent models and, most importantly, psychiatric patients.

Although systemic MK0677 administration induced an eating response in S1 as well as BL6 mice strains, it did not attenuate the extinction-impaired phenotype of S1 mice. This finding is in line with our results from experiments in extinction-competent BL6 mice, in which neither systemic nor central (ventral tegmental area) injection of MK0677 had an effect on fear extinction (29). Other authors, however, previously reported that MK0677 reduced fear memory strength in unstressed rats and enhanced fear extinction C57BL/6 mice after systemic or intra-amygdalar application (14, 19). MK0677 doses in our experiments (2–10 mg/kg) were in the range other studies reporting central drug effects used for i.p. administration in rodents (14, 43, 46). Moreover, these doses were already associated with a strong instatement of hunger, as shown here and in our previous study (29), which could potentially confound behavioral readouts. Thus, we do not believe that testing higher doses would have experimental or translational value. While only a few studies are published which tested behavioral effects of small molecule GHSR agonists like MK0677, there are abundant reports of anxiolytic, fear-attenuating and antidepressant effects of ghrelin peptide administration (13, 15–17, 47). Other studies contradict these findings and some even suggest anxiogenic or fear-enhancing effects after peripheral or central administration of acyl ghrelin (43, 48–51). Interestingly, most studies that reported a beneficial effect of GHSR agonism on fear-, anxiety-, and depressive-like behaviors were conducted in previously stress-exposed rodents, while findings in unstressed rodents were more mixed [for summary see (6)]. Thus, it seems likely that the effect of GHSR agonists may depend on various (experimental) factors like previous stress exposure or also handing routines and different behavioral paradigms, which *per se* could be perceived as more or less stressful by the tested animals. Other reasons for the inconsistent findings could involve differences in age, sex and strain of the used rodent models, various administration routes (e.g., central vs. peripheral), the time of day of testing and also the feeding status of the animals (6).

As an alternative to GHSR agonism, we studied whether overnight fasting (as a method to enhance endogenous ghrelin signaling) would rescue the extinction-impaired phenotype of S1 mice. Several studies have demonstrated that caloric restriction or short-term fasting strongly facilitate within-session fear extinction and extinction memory formation in extinction-competent mice (18–20) or reduce anxiety-like behaviors (13, 21) *via* different mechanisms, but probably also in a GHSR-dependent manner (13, 19, 21). In contrast, we previously found no effect of overnight fasting on fear extinction or extinction retrieval in BL6 or GHSR-KO mice (29). Also, in S1 mice, overnight fasting did not influence within-session extinction or extinction memory consolidation. In the fasted state, S1 mice even showed higher freezing levels than non-fasted controls, while in extinction retrieval sessions, when all animals were fed, there was no significant difference in the freezing behavior of both treatment groups. This suggests that that the stressful nature of an overnight fast may have had adverse effects in this anxiety-prone mouse strain (52), which resolved after re-feeding. The fact that we could not confirm the reports of a beneficial effect of fasting in unstressed rodents raises the question to which experimental parameters this effect may be susceptible and whether it is robust enough to translate into clinical studies. A recent meta-analysis found that fasting or caloric restriction decreased anxiety, stress and depression levels in a few small population-based studies (53) and one study in healthy human participants reported that short-term fasting improved extinction retention and reduced return of fear phenomena, which was correlated with plasma ghrelin levels (22). However, while these preliminary results may seem encouraging, they do not form a solid foundation to recommend fasting as a suitable treatment option at this point. To our best knowledge, no successful clinical trial of a fasting intervention in anxiety patients has been conducted to date. Also, with regard to the translational value of fasting experiments, it should be considered that forced fasting of experimental animals, who do not comprehend the context of their distress, and controlled food restriction within the framework of a clinical study may not deliver comparable results.

In summary, our data suggest that an activation of the ghrelin system does not facilitate fear extinction (in unstressed individuals), but underline the possibility that elevated acyl ghrelin could be a biomarker for fear-susceptible and extinction-impaired individuals. With regard to the divergent reports of ghrelin’s role in stress and mood regulation (6), our results highlight once more the need to investigate which factors may influence the effects of fasting and GHSR agonism on fear- and anxiety-like behaviors in order to better understand the consequences of (therapeutically) manipulating the ghrelin system and guarantee the greatest benefit for patients from future clinical trials.

## Data availability statement

The raw data supporting the conclusions of this article will be made available by the authors, without undue reservation.

## Ethics statement

This animal study was reviewed and approved by Austrian Federal Ministry of Science and Research.

## Author contributions

NS and DD conceptualized and overviewed the project. EF performed all experiments and data analysis with methodological input from AP. EF wrote the manuscript. DD and NS reviewed and revised all sections. All authors read and approved the final manuscript.
