# Supplementary material for: Ghrelin receptor agonist MK0677 and overnight fasting do not rescue deficient fear extinction in 129S1/SvImJ mice
Source: Front Psychiatry. 2023 Feb 9;14:1094948. doi: 10.3389/fpsyt.2023.1094948 (PMC9947350; doi:10.3389/fpsyt.2023.1094948)
Supplement: Supplementary file 1 [file Data_Sheet_1.PDF]

Supplementary Material

1 Supplementary Methods

1.1 Total and acyl ghrelin ELISA assays - Assay characteristics according to manufacturer

Analytical sensitivity: The lowest level of active ghrelin that can be detected by this assay (*#EZRGRA-90K*) is 8 pg/mL when using a 20 µL sample size. The lowest level of total ghrelin that can be detected by this assay is 0.04 ng/mL when using a 20 µL sample size.

Specificity: rat/mouse ghrelin (active) 100% (*#EZRGRA-90K*), 85% (*#EZRGRA-91K*); des-octanoyl rat/mouse ghrelin 0% (*#EZRGRA-90K*), 100% (*#EZRGRA-91K*)

Intra- and inter-assay variability (#EZRGRA-90K, #EZRGRA-91K): Plasma samples from six animals were pooled and treated with AEBSF and HCl, then divided into 3 aliquots. Various amounts of rat/mouse (des-octanoyl) ghrelin were added to the aliquots to create low, intermediate and high levels of ghrelin samples for precision tests. Intra-assay variations were calculated from results of six duplicate determinations in one assay. Inter-assay variations were calculated from results of six separate assays with duplicate samples in each assay.

Sample	Active ghrelin (pg/mL) mean, n = 6	Intra-assay CV (%)	Inter-assay CV (%)
Mouse plasma low	58	5.56	4.70
Mouse plasma interm.	210	2.70	4.08
Mouse plasma high	1035	0.86	1.23

Sample	Total ghrelin (ng/mL) mean, n = 6	Intra-assay CV (%)	Inter-assay CV (%)
Mouse plasma low	1.84	1.56	2.90
Mouse plasma interm.	3.13	1.07	2.92
Mouse plasma high	4.89	1.07	2.81

1.2 Fear conditioning and extinction – Detailed protocols

Fear conditioning (FC, context A): The conditioning chamber was a 25 x 25 x 35 cm cubicle with transparent walls, an open top and a metal rod floor that was cleaned with water and illuminated with white light at 300 lux. After a 120 s acclimation period, five pairings of a CS (10 kHz sine tone, 75 dB, 30 s) with a co-terminating US (scrambled foot shock, 0.6 mA, 2 s) were delivered with a 120 s inter-tone interval. After the final pairing, a 120 s no-stimulus consolidation period followed, before the mice were returned to their home cage.

Fear extinction training (EXT, context B): The extinction context was a 25 x 25 x 35 cm cubicle with black walls, an open top and a solid grey floor that was cleaned with 1% acetic acid and illuminated

with red light to 5 lux. After a 120 s acclimation period, 25 CS presentations with a 5 s inter-tone interval were delivered. After the final presentation, a 120 s no-stimulus consolidation period followed, before the mice were returned to their home cage.

Extinction retrieval (RET, context B): For extinction retrieval, the mice were again placed in the extinction context (context B) 24 h following extinction training. After a 120 s acclimation period, 5 CS presentations with a 5 s inter-tone interval were delivered. After the final presentation, a 120 s no-stimulus consolidation period followed, before the mice were returned to their home cage.

Fear reinstatement (REIN, context A/B): For fear reinstatement, the mice were again placed in context A two weeks following the last extinction retrieval session. After 120 s, an unsignaled US was delivered. Then, a 120 s no-stimulus consolidation period followed before the mice were returned to their home cage. Fear reinstatement was tested 24 h later in context B with a presentation of 5 CS.

2 Supplementary Tables

Figure	Mann-Whitney Test	S1 saline vs. MK0677	BL6 saline vs. MK0677
1A	food intake (home cage)	p=0.8785	p=0.7789
1B	baseline – food intake	p=0.8785	p=0.6126
1C	baseline – time spent eating	p=0.0769	p=0.4615
1D	baseline – distance travelled	p>0.9999	p=0.6943

Supplementary Table 1. *Statistical comparison of S1 and BL6 saline and MK0677 groups at baseline.* For graphs, overnight and baseline data were pooled per strain, as there was no significant difference between the groups pre-treatment.

Figure	Statistical test	p	t, MWU	df, rank sums
1A	Unpaired Student's t-test	p=0.9580	t=0.0531	df=29
1B	Mann-Whitney test	p=0.0021	MWU=44.50	180.5 (S1)<br>315.5 (BL6)
1C	Mann-Whitney test	p=0.0001	MWU=33	169 (S1)<br>327 (BL6)
1D	Mann-Whitney test	p=0.0023	MWU=45	181 (S1)<br>315 (BL6)
3B, B2	Unpaired Student's t-test	p=0.5405	t=0.6273	df=14
3B, B4	Unpaired Student's t-test	p=0.9252	t=0.0957	df=14
3B, B6	Unpaired Student's t-test	p=0.9517	t=0.0616	df=14
3B, B7	Unpaired Student's t-test	p=0.5134	t=0.6706	df=14
4B, B2	Unpaired Student's t-test	p=0.3538	t=0.9370	df=45
4B, B4	Unpaired Student's t-test	p=0.8468	t=0.1943	df=45
4B, B6	Unpaired Student's t-test	p=0.7210	t=0.3594	df=45
4B, B7	Unpaired Student's t-test	p=0.4150	t=0.8227	df=45

Supplementary Table 2. *Overview of p-values, t-values and df for Student's t-tests or Mann-Whitney U (MWU) and rank sums for Mann-Whitney tests.*

Figure	Statistical test	p	F (DFn, DFd)
1E	Two-way ANOVA	'treatment' p<0.0001<br>'strain' p=0.5853<br>'interaction' p=0.5210	F (1, 27) = 40.04<br>F (1, 27) = 0.3051<br>F (1, 27) = 0.4229
1F	Two-way ANOVA	'treatment' p=0.0018<br>'strain' p=0.1342<br>'interaction' p=0.3335	F (1, 27) = 11.98<br>F (1, 27) = 2.384<br>F (1, 27) = 0.9696
1G	Two-way ANOVA	'treatment' p=0.1492<br>'strain' p=0.6712<br>'interaction' p=0.5803	F (1, 27) = 2.205<br>F (1, 27) = 0.1841<br>F (1, 27) = 0.3133
1H	Two-way RM ANOVA	'stage' p<0.0001<br>'strain' p=0.2517<br>'interaction' p=0.9554	F (1, 13) = 35.08<br>F (1, 13) = 1.439<br>F (1, 13) = 0.0033
1I	Two-way RM ANOVA	'stage' p=0.0035<br>'strain' p=0.1271<br>'interaction' p=0.5048	F (1, 13) = 12.70<br>F (1, 13) = 2.657<br>F (1, 13) =0.4706
1J	Two-way RM ANOVA	'stage' p=0.6435<br>'strain' p=0.1221<br>'interaction' p=0.0479	F (1, 13) = 0.2245<br>F (1, 13) = 2.735<br>F (1, 13) = 4.769
2A	Two-way ANOVA	'treatment' p<0.0001<br>'strain' p=0.6910<br>'interaction' p=0.5937	F (1, 28) = 25.52<br>F (1, 28) = 0.1613<br>F (1, 28) = 0.2912
2B	Two-way ANOVA	'treatment' p<0.0001<br>'strain' p=0.0097<br>'interaction' p=0.7551	F (1, 28) = 34.72<br>F (1, 28) = 7.716<br>F (1, 28) = 0.0993
2C	Two-way ANOVA	'treatment' p=0.9017<br>'strain' p=0.0098<br>'interaction' p=0.5564	F (1, 28) = 0.0155<br>F (1, 28) = 7.672<br>F (1, 28) = 0.3544
3B, A1	Two-way RM ANOVA	'treatment' p=0.9588<br>'CS block' p<0.0001<br>'interaction' p=0.3055	F (1, 14) = 0.0028<br>F (3.537, 49.52) = 27.27<br>F (4, 56) = 1.238
3B, B1	Two-way RM ANOVA	'treatment' p=0.5834<br>'CS block' p=0.1546<br>'interaction' p=0.8033	F (1, 14) = 0.3152<br>F (3.070, 42.98) = 1.832<br>F (4, 56) = 0.4063
3B, B3	Two-way RM ANOVA	'treatment' p=0.1859<br>'CS block' p=0.0019<br>'interaction' p=0.9600	F (1, 14) = 1.935<br>F (2.445, 34.23) = 6.824<br>F (4, 56) = 0.1548
3B, B5	Two-way RM ANOVA	'treatment' p=0.3506<br>'CS block' p<0.0001<br>'interaction' p=0.3425	F (1, 14) = 0.9324<br>F (2.786, 39.01) = 13.95<br>F (4, 56) = 1.151
4B, A1	Two-way RM ANOVA	'treatment' p=0.1920<br>'CS block' p<0.0001<br>'interaction' p=0.0678	F (1, 45) = 1.755<br>F (3.542, 159.4) = 56.56<br>F (4, 180) = 2.227
4B, B1	Two-way RM ANOVA	'treatment' p=0.0011<br>'CS block' p=0.0008<br>'interaction' p=0.0854	F (1, 45) = 12.11<br>F (2.916, 131.2) = 6.062<br>F (4, 180) = 2.079
4B, B3	Two-way RM ANOVA	'treatment' p=0.0346	F (1, 45) = 4.749

		‘CS block’ p<0.0001<br>‘interaction’ p=0.4066	F (3.290, 148.0) = 7.862<br>F (4, 180) = 1.004
4B, B5	Two-way RM ANOVA	‘treatment’ p=0.0052<br>‘CS block’ p<0.0001<br>‘interaction’ p=0.2765	F (1, 45) = 8.618<br>F (3.495, 157.3) = 7.784<br>F (4, 180) = 1.288

Supplementary Table 3. ***Overview of p-values and F-values (DFn, DFd) for Two-way (RM) ANOVAs.***

3 Supplementary Figures

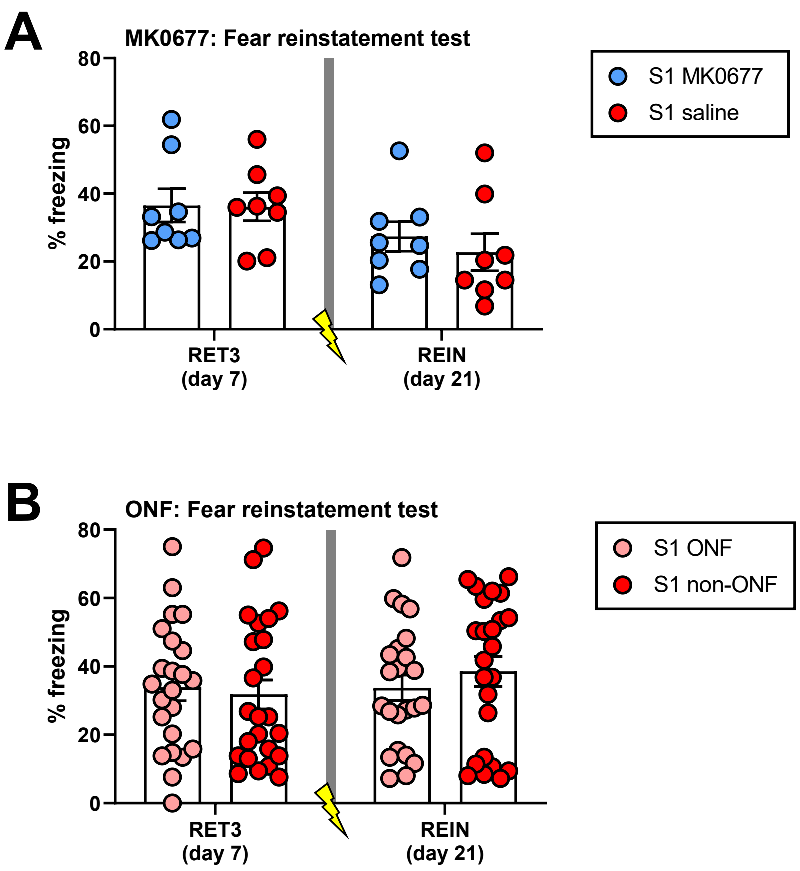

Supplementary Figure 1. ***Freezing levels in MK0677- and saline-treated (A) or overnight fasted (ONF) or non-fasted (B) S1 mice during the last extinction retrieval session (RET3) and fear reinstatement test (REIN).*** **A.** MK0677- and saline-treated S1 mice did not show a significant increase in freezing levels following the delivery of an unsignaled foot shock in context A two weeks after extinction training. **B.** Overnight fasted and non-fasted S1 mice did not show a significant increase in freezing levels following the delivery of an unsignaled foot shock in context A two weeks after extinction training.. *Figure info:* S1 MK0677 (blue): n = 8; S1 saline (red): n = 8; S1 ONF (pink): n = 23; S1 non-ONF (red): n = 24. All data points are individual values and all bars represent group means ± SEM for 5-CS average blocks
